# Supplementary material for: Dysregulation of the NUDT7-PGAM1 axis is responsible for chondrocyte death during osteoarthritis pathogenesis
Source: Nat Commun. 2018 Aug 24;9:3427. doi: 10.1038/s41467-018-05787-0 (PMC6109082; doi:10.1038/s41467-018-05787-0)
Supplement: Supplementary file 1 — Supplementary Information [file 41467_2018_5787_MOESM1_ESM.pdf]

Supplementary Information

Dysregulation of the NUDT7-PGAM1 axis is responsible for chondrocyte death during osteoarthritis pathogenesis

Supplementary Figure 1. Peroxisomal dysfunction in osteoarthritis (OA) pathogenesis. (a) Basic information of OA patient for analysis of peroxisomal function. (b and c) Analysis of glutathione peroxidase (GPx) and catalase activity (n = 11 per group). (d) Immunocytochemistry of 70-kDa peroxisomal membrane protein (PMP70) and β-oxidation (n = 5 per group). (e) Transcript levels of matrix synthesis genes in chondrocytes from patients with OA compared with those in normal chondrocytes (n = 8). (f) Levels of free fatty acid in OA chondrocytes compared those in normal chondrocytes (n = 6). Scale bars, 10 μm. Values are means + s.d. A unpaired Student's t test was used for statistical analysis. *P < 0.05, **P < 0.01, ***P < 0.001, ****P < 0.0001.

Supplementary Figure 1

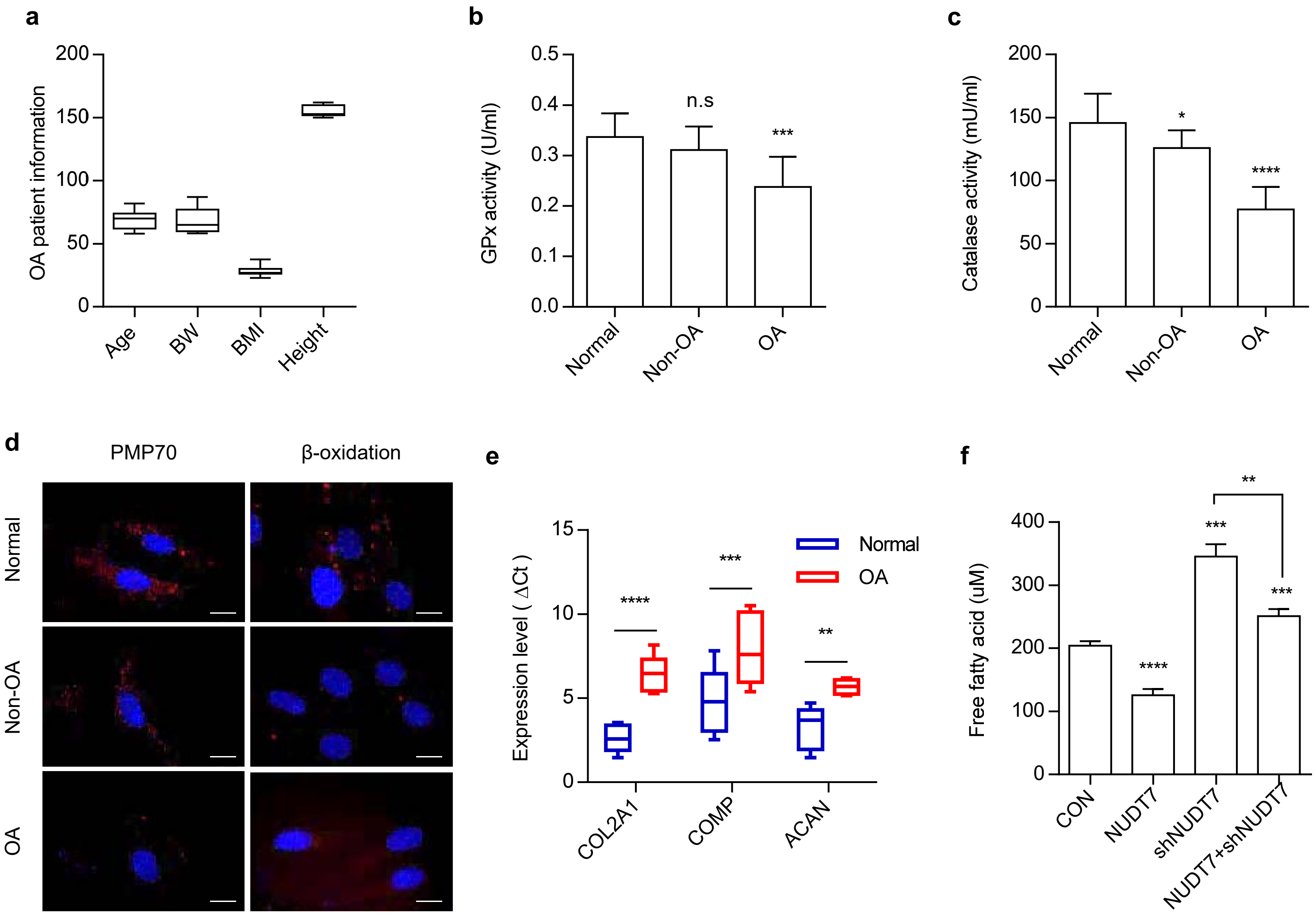

Supplementary Figure 2. *NUDT7* is responsible for dysregulation of lipid metabolism and peroxisome. Staining of lipid reactive oxygen species (ROS), lipid accumulation (BODIPY$^{493/508}$), β-oxidation, and 70-kDa peroxisomal membrane protein (PMP70) in normal chondrocytes infected with lentivirus containing *NUDT7* or *NUDT7* shRNA (*shNUDT7*) (n=5). Scale bar, 10 μm.

Supplementary Figure 2

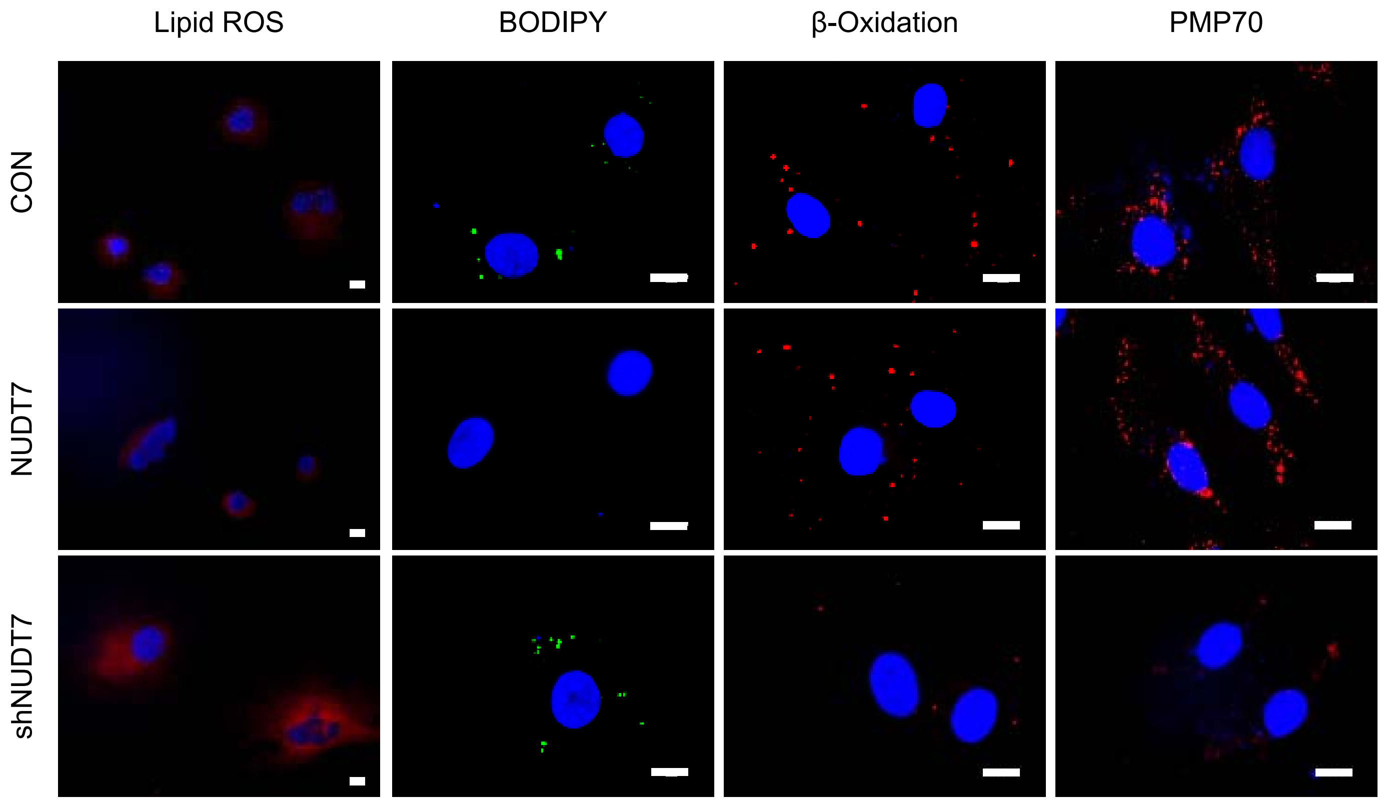

Supplementary Figure 3. Spontaneous cartilage degradation induces in *Nudt7*$^{-/-}$ mice. Staining with Safranin O, and for Collagen C1-2C and matrix metalloproteinase (MMP)-13 and counting of Collagen C1-2C and MMP-13-positive-cells. Scale bars, 50 μm. Values are means + s.d. A unpaired Student's t test was used for statistical analysis. **P < 0.01, ****P < 0.0001.

Supplementary Figure 3

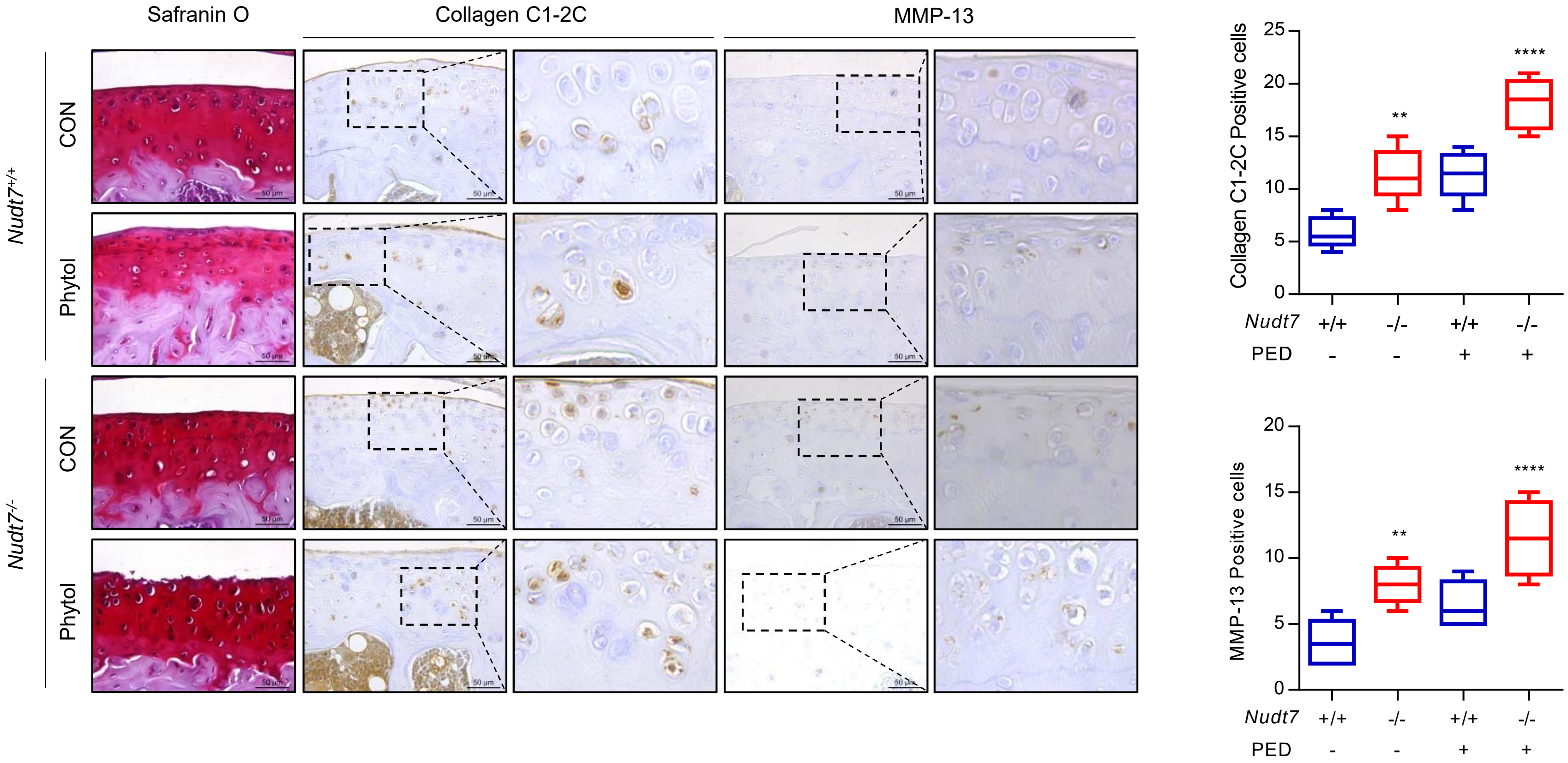

Supplementary Figure 4. Peroxisome dysfunction in *Nudt7*$^{-/-}$ mice. (a) Transcription levels of collagen type 2A1(*COL2A1*) in iMACs of *Nudt7*$^{-/-}$ mice at postnatal day 6 under 50 μM phytol conditions compared to those of *Nudt7*$^{+/+}$ mice (n = 3 per group). (b) Analysis of cell proliferation in iMACs of *Nudt7*$^{-/-}$ mice at postnatal day 6 under 50 μM phytol conditions compared to those of *Nudt7*$^{+/+}$ mice (n = 3 per group). (c and d) Activities of catalase and glutathione peroxidase (GPx) in in iMACs of *Nudt7*$^{-/-}$ mice at postnatal day 6 under 50 μM phytol conditions compared to those of *Nudt7*$^{+/+}$ mice (n = 5 per group). Values are means + s.d. A unpaired Student's t test was used for statistical analysis. **P < 0.01, ****P < 0.0001.

Supplementary Figure 4

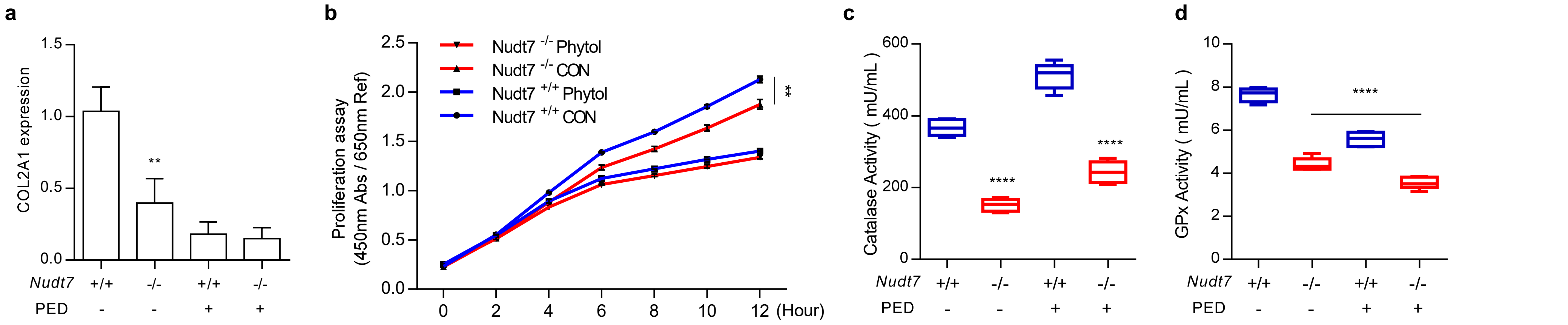

Supplementary Figure 5. Mitochondrial dysfunction occurs in osteoarthritis (OA) chondrocytes via glycolysis overload. (a) Measurement of mitochondrial membrane potential using Muse Mitopotential Assay Kit in iMACs of *Nudt7*$^{-/-}$ mice at postnatal day 6 under 50 μM phytol conditions compared to those of *Nudt7*$^{+/+}$ mice (n = 6 per group). (b) Analysis of pyruvate accumulation in iMACs of *Nudt7*$^{-/-}$ mice at postnatal day 6 under 50 μM phytol conditions compared to those of *Nudt7*$^{+/+}$ mice (n = 5 per group). (c) Immunostaining of fatty acid synthase (FASN) in human OA chondrocytes compared with that in normal chondrocytes (n = 5). Values are means + s.d. A unpaired Student's t test was used for statistical analysis. ***P < 0.001, ****P < 0.0001.

Supplementary Figure 5

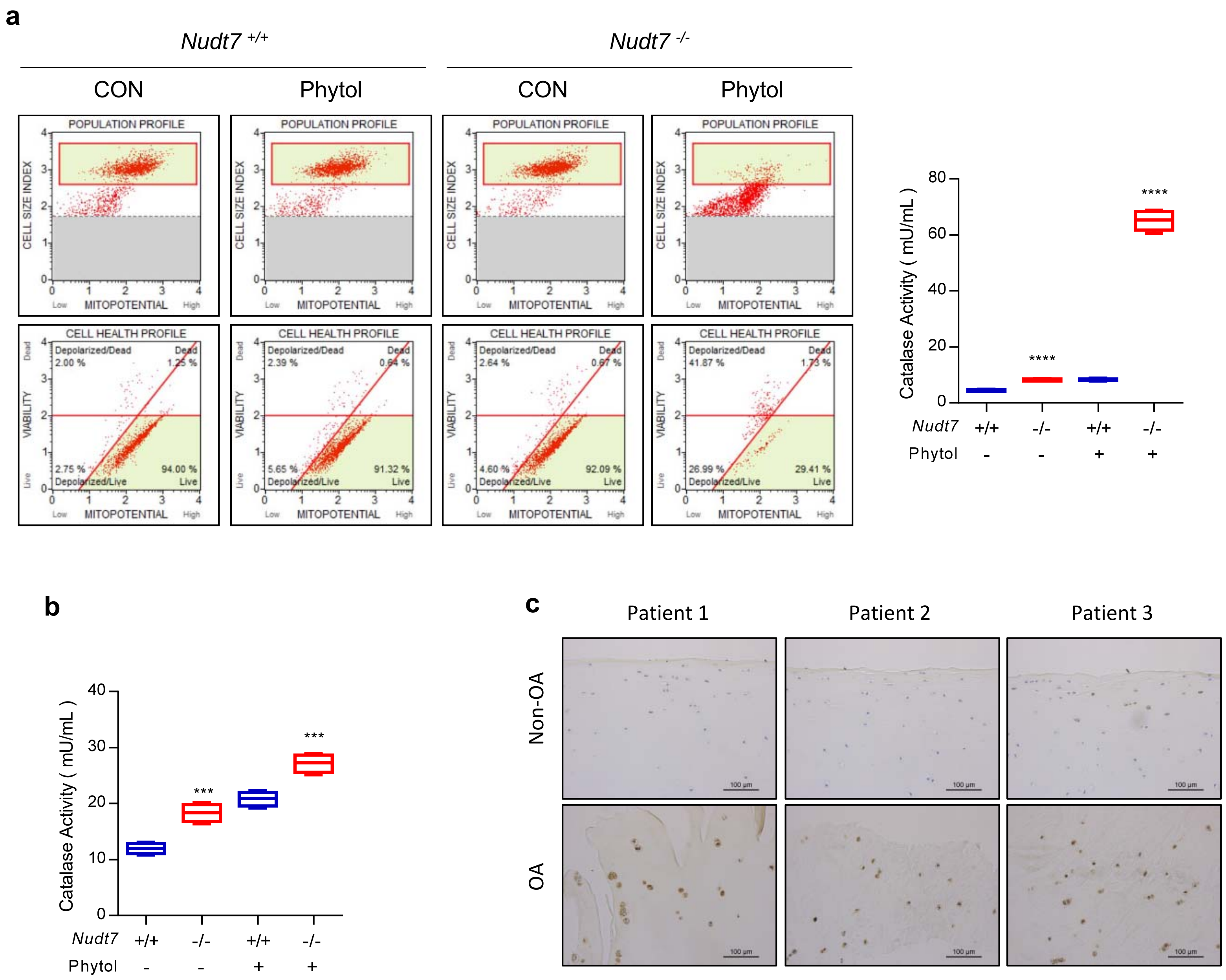

Supplementary Figure 6. Common GSEA (Gene Set Enrichment Analysis) pathway analysis between human osteoarthritis (OA) chondrocytes and the *Nudt7*$^{-/-}$ mice chondrocyte. The total 11 GSEA pathways were merged in human OA chondrocyte and *Nudt7*$^{-/-}$ mice chondrocyte.

Supplementary Figure 6

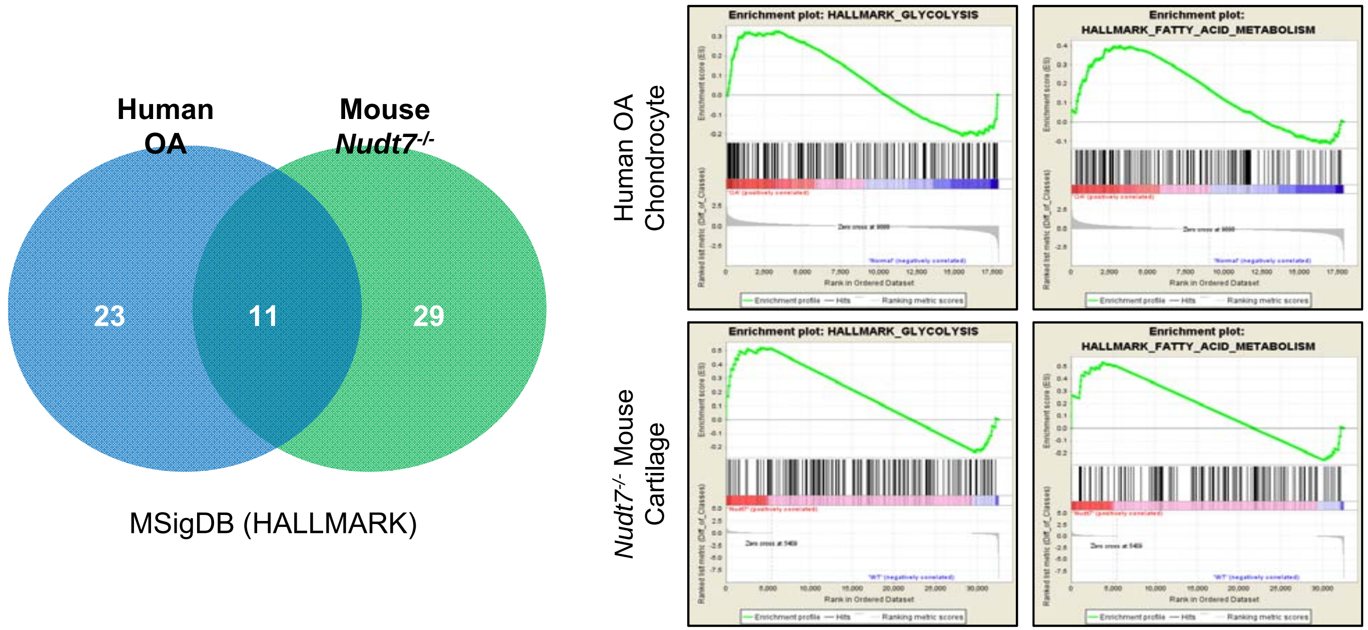

Top 5 of Human OA Chondrocyte	ES	NES	NOM p-val
HALLMARK_DNA_REPAIR	0.333	1.236	0.075
HALLMARK_GLYCOLYSIS	0.329	1.260	0.050
HALLMARK_HEDGEHOG_SIGNALING	0.455	1.336	0.080
HALLMARK_FATTY_ACID_METABOLISM	0.400	1.483	0.010
HALLMARK_MYC_TARGETS_V2	0.644	2.095	0.000

Top 5 of Nudt7-/- Mouse Chondrocyte	ES	NES	NOM p-val
HALLMARK_ANGIOGENESIS	0.709	1.116	0.313
HALLMARK_BILE_ACID_METABOLISM	0.544	1.012	0.447
HALLMARK_GLYCOLYSIS	0.523	1.040	0.370
HALLMARK_E2F_TARGETS	0.469	0.935	0.582
HALLMARK_FATTY_ACID_METABOLISM	0.537	1.021	0.408

Supplementary Figure 7. The inflammatory response is upregulated in the cartilage of *Nudt7*$^{-/-}$ mice. Transcript levels of inflammatory cytokines and receptors were determined and applied to Ingenuity Pathway Analysis (IPA)

Supplementary Figure 7

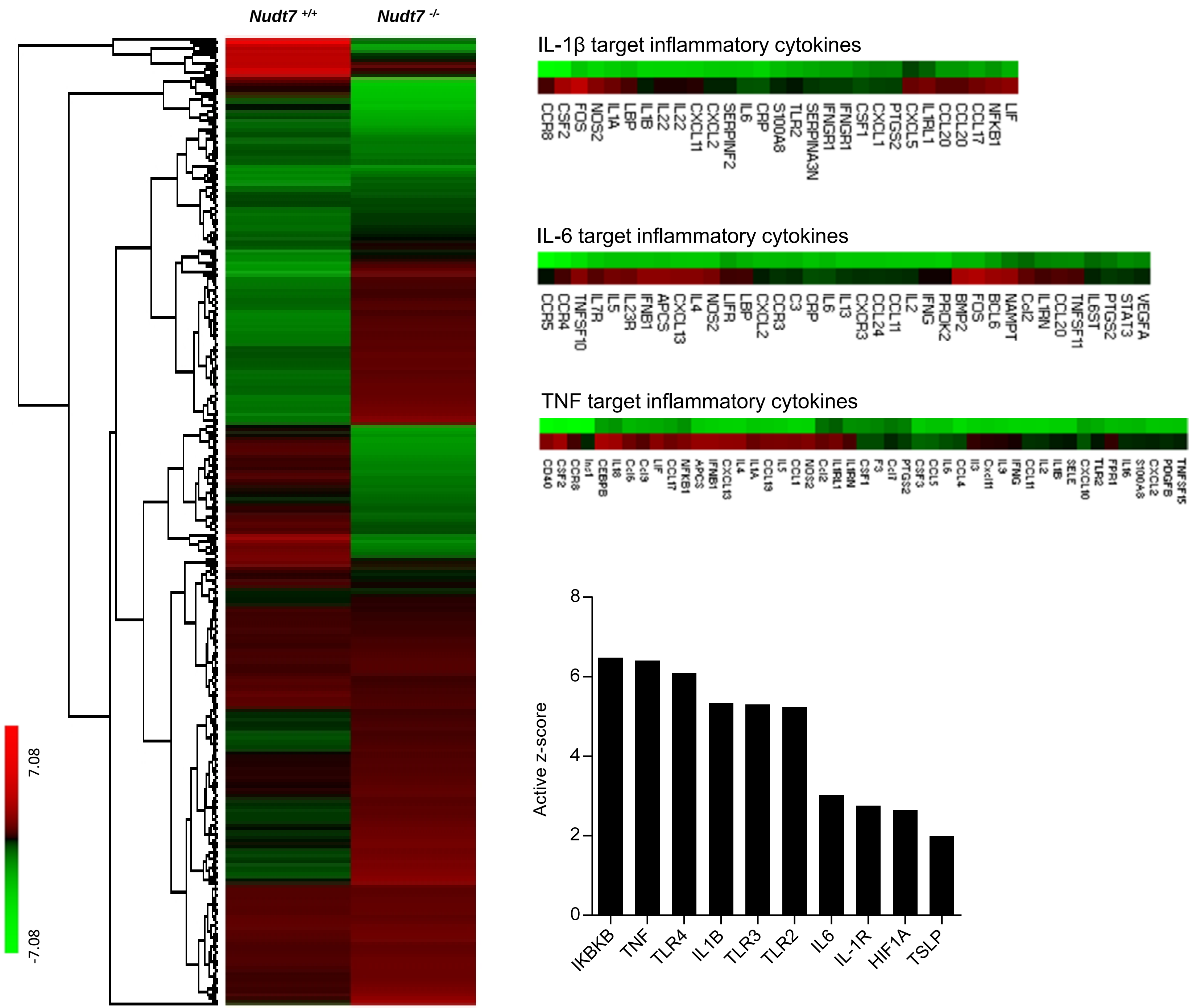

Supplementary Figure 8. Histone modification is involved in the osteoarthritis (OA) pathogenesis. Immunocytochemistry of H3K27me3 and counting of H3K27me3-positive cells among immature murine articular chondrocytes (iMACs) of *Nudt7*$^{-/-}$ mice compared to iMACs of *Nudt7*$^{+/+}$ mice (n = 6 per group). Scale bars 200 μm. Values are means + s.d. A unpaired Student's t test was used for statistical analysis. **P < 0.01.

Supplementary Figure 8

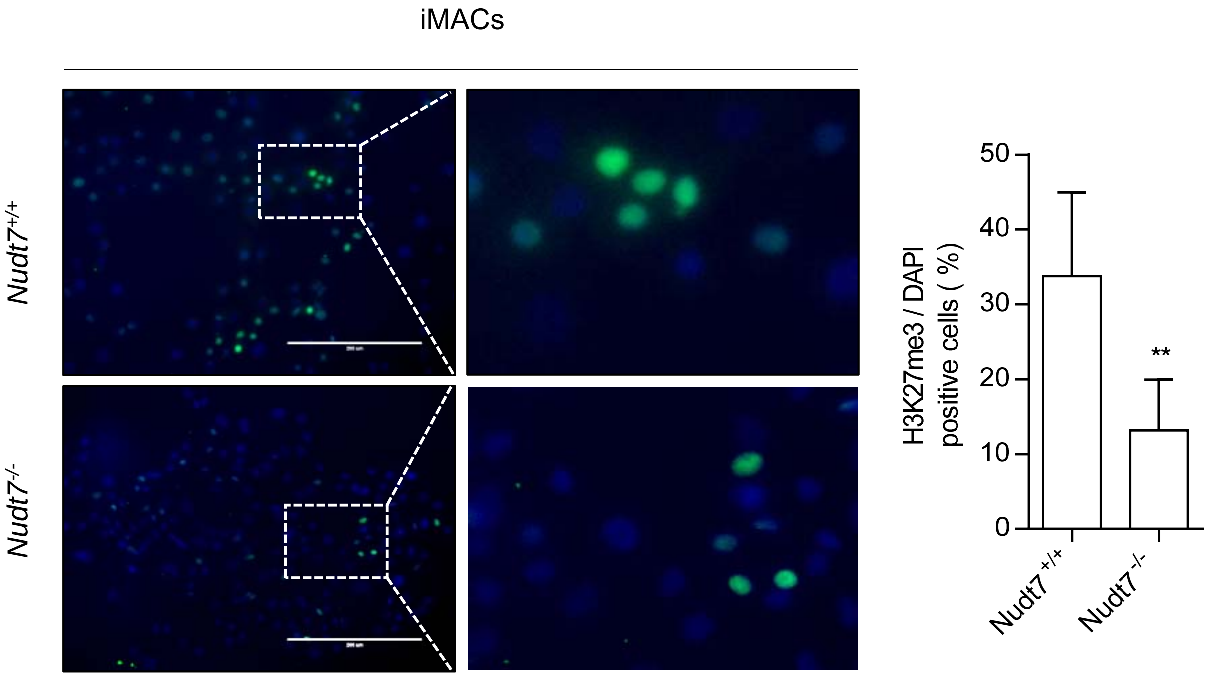

Supplementary Figure 9. Recovery of phosphoglycerate mutase 1 (PGAM1)-induced cartilage degradation by knockdown of *PGAM1*.

Supplementary Figure 9

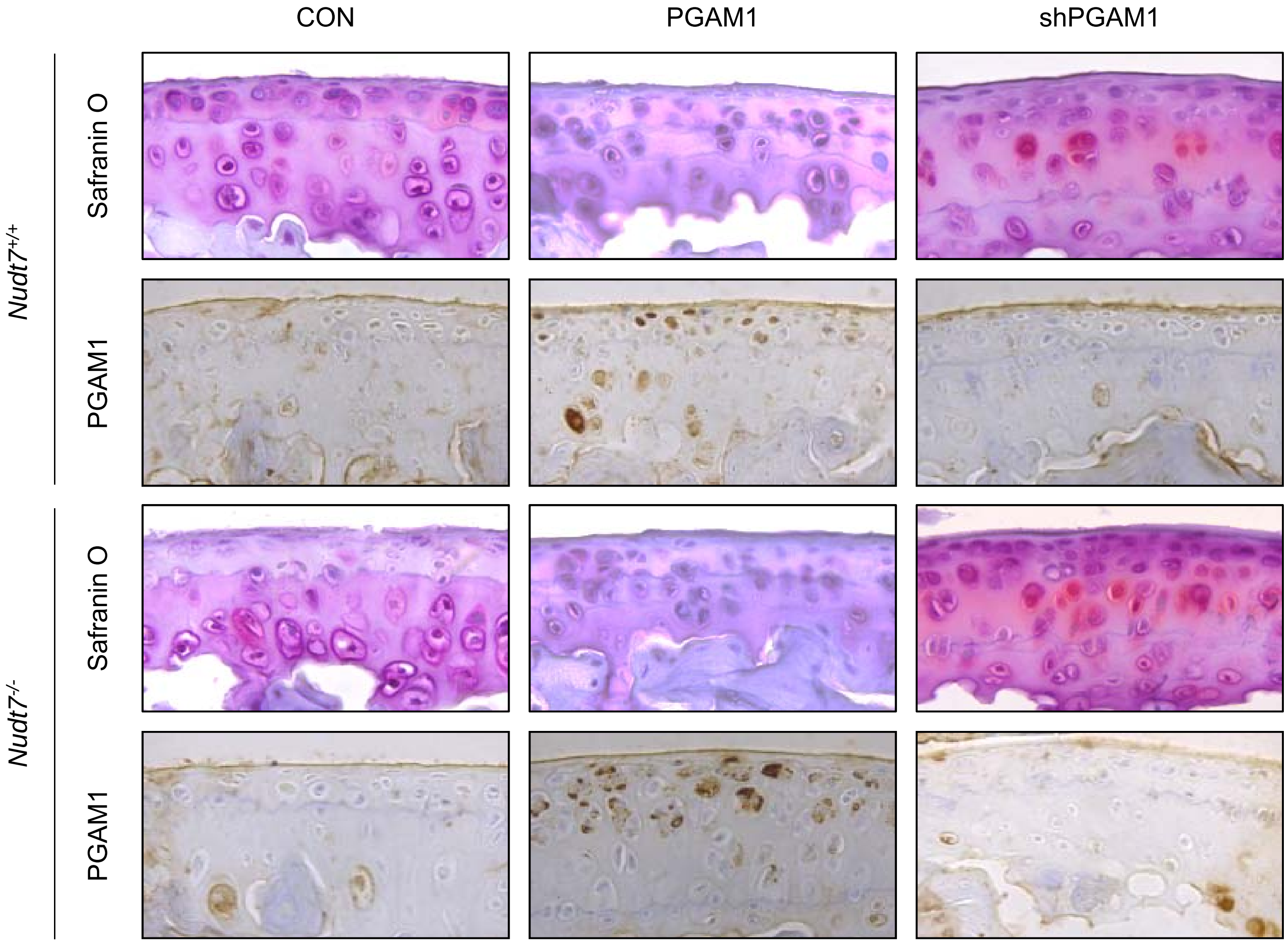

Supplementary Figure 10. Introduction of *NUDT7* suppresses *PGAM1*-induced cartilage degradation. Cartilage degradation was measured using Mankin score and OARSI score (n = 6 per group). Scale bar = 50 μm. Values are means + s.d. Values are means + s.d. A unpaired Student's t test was used for statistical analysis. ***P < 0.001, ****P < 0.0001.

Supplementary Figure 10

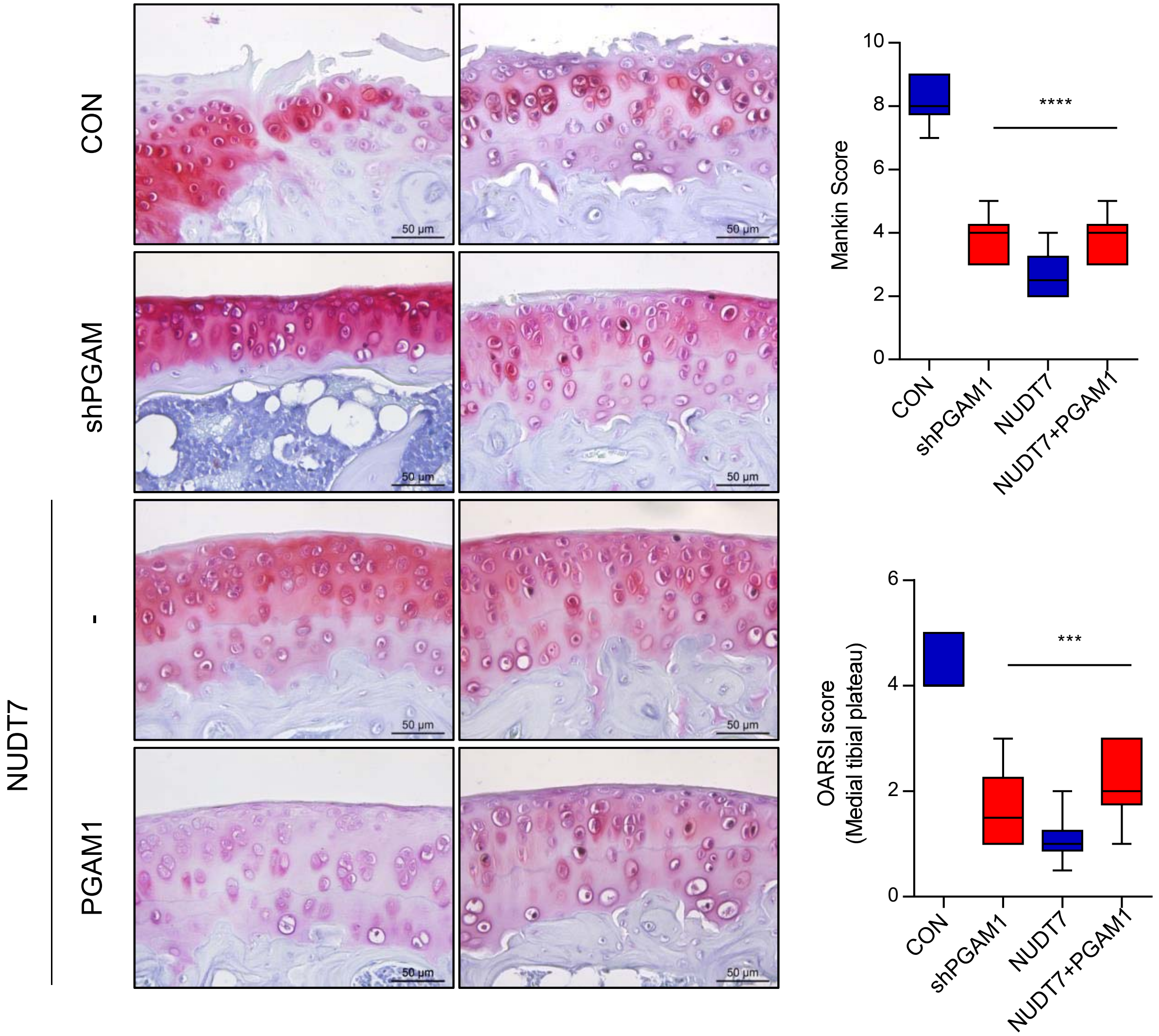

Supplementary Figure 11. The effect of *NUDT7* in skeletal growth and trabecular bone formation. (a) Safranin O staining in E18.5 *Nudt7*$^{-/-}$ mice compared *Nudt7*$^{+/+}$ mouse (n = 5 per group) and hypertrophic zone was measured. (b) Alizarin red/Alcian blue staining of a newborn skeleton of limb in *Nudt7*$^{-/-}$ and *Nudt7*$^{+/+}$ mice (n = 8 per group) and size of femur and tibia were measured. (c and d) microCT analysis of trabecular bone in *Nudt7*$^{-/-}$ and *Nudt7*$^{+/+}$ mouse (n = 4 per group).

Supplementary Figure 11

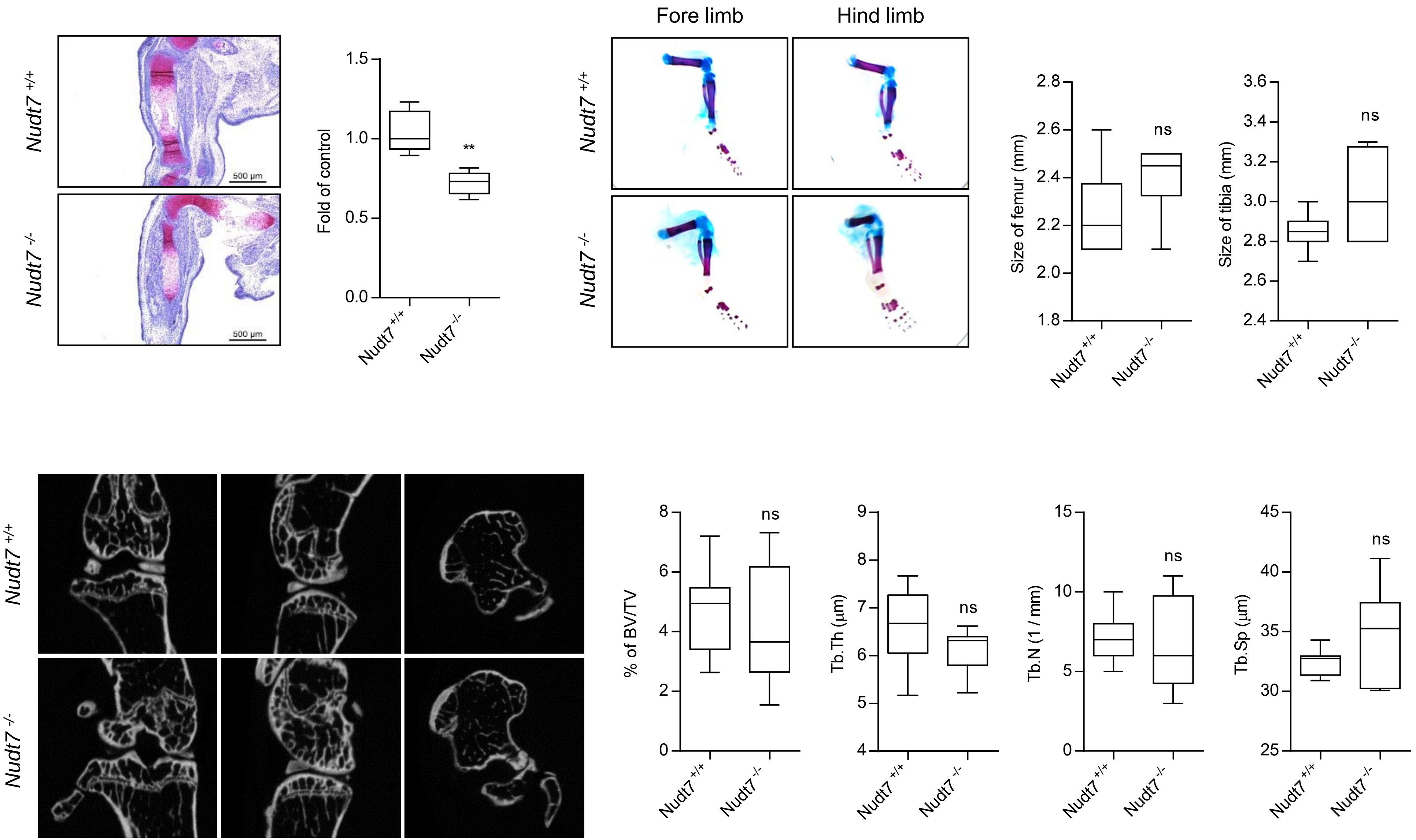

Supplementary Figure 12. Increased level of fatty acid induced the level of IL-1β and MMP-13. Transcript levels of IL-1β and MMP-13 in OA chondrocytes introduced with/without *NUDT7* in the presence of 50 μM palmitic acid (PA) or linoleic acid (LA) (n = 3 per group). Values are means + s.d. A unpaired Student's t test was used for statistical analysis. **P < 0.01.

Supplementary Figure 12

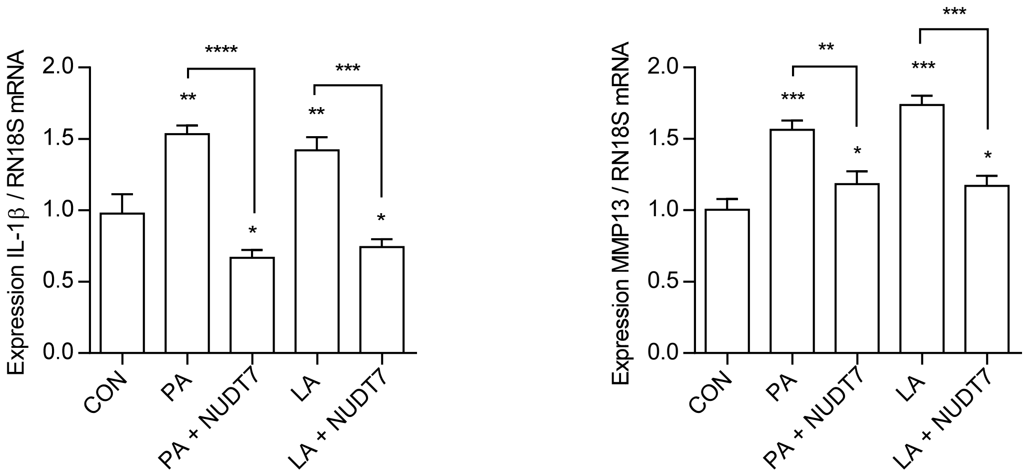

Supplementary Figure 13. Uncropped image of the western blots related to Figure 4d.

Supplementary Figure 13

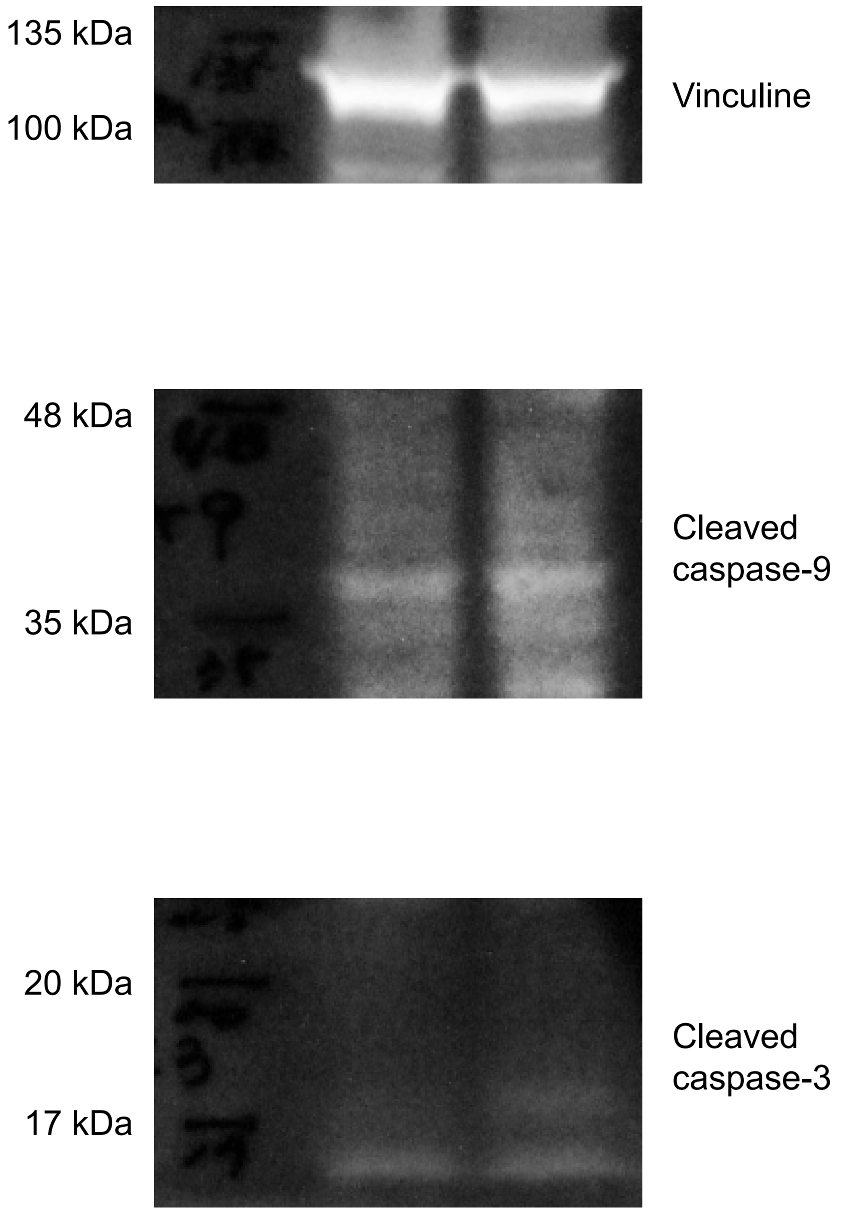

Supplementary Table 1. Human qRT-PCR primers for analyzing the expression levels of peroxisome related genes.

Supplementary Table 1

Human peroxisome related genes	Forward primer sequence	Reverse primer sequence
ABCD1	ATGCAAAGGAAGGGCTACTC	CGTCCTTCCAGTCACACATAG
ABCD2	AAGAGAAGGAGGATGGGATG	GGTACATTCATCCAGCAAGG
ABCD3	TGTCAGTCGCCCTTTCTTAG	CTATTCGACCCAGAGCTTGA
ABCD4	TGAGCATCTTCGGGTATTTC	TCTGCATGTGCTTGAACCTA
ACAA1	TAGCAGGTGGCATCAGAAATG	CTCCATCAAGCGCGAAGTAATA
ACAD11	GCTGGTGTTTCCCAGGATTA	CAGCTCCATTGTTGCGATTG
ACBD5	AATTGCTGCGTGTCATAGGT	TTTGGCGTTTGGAGTAGAAG
ACOT1	GGTGACCAAAGATGGCTATG	TGACCTACCAGGAACAGGAA
ACOT12	AGCACAAGCATGGAGATCAG	GTGGAGAAAGCCACACTAACA
ACOT2	CTTGGTGGGCAGTCCTATTATC	CCCAAGTGTTTGTGGAAGAAAG
ACOT4	CCACGTTGGCTCTAGCTTATT	GCATGTAGCATACGGCTTCT
ACOT8	GGACCCTAACCTCCAAAAGA	ATCTGTTTGGGCTCCATTCT
ACOX1	CAGGAATTACCGTTGGTGAC	TTCACCTGGGCATACTTCAT
ACOX2	ACATGGCAAGAACAGCCTAC	TCATAACAGCCAAGTGCTGA
ACOX3	TTGCTCTGACCGAATTAAGC	CGAAATCAGGGGAATGTATG
ACSF3	ACACGTACAGGGAGCTTTATTC	GTTAGCGCATAGGAAGGAGAC
ACSL1	ACGAAGATCCGCACTACTTG	CAAGGGCCATTATTTGACAC
ACSL3	AAGCTTGCTAGGGGGAAATA	ACCAACAGGACAGCAGAAAC
ACSL4	GCAGAGTACCCTGAAGGATTTG	CGTTGGTCTACTTGGAGGAATG
ACSL5	TACCTGGGTTCCTGTCTCTT	GATGATCCACTCTGGCCTATTC
ACSL6	TATCCGCCTTCTCTCAGATG	CTGGCTGAAGATCTTGTCGT
ACTB	AGGCACCAGGGCGTGAT	GCCCACATAGGAATCCTTCTGAC
AGPS	GTTGGCAGCTGGAGAAGATA	CCAAGGAGCAGAAGTCTCAA
AGXT	GGTCCATGAGCAAGGATATGT	CAGAGATGACCAGTGTGAGTG
ALDH3A2	CATGCTGGATGAGGCCTATATT	AGTGGCTGAATGGTGAGAAC
AMACR	GCTGGCCACGATATCAACTAT	CAAAGTCAGCCAGGAGATTCA
BAAT	GCTGAAGAGACATGGGAAGAA	GCACAGCACAGAGGAGAATAG
CAT	AATCCATTCGATCTCACCAA	GGTCGAAGGCTATCTGTTCA
CRAT	AGACAAGGTGAACCGGGATTCCG	GGCTGCGGTACACGTCTTCTGA
CROT	GCACTTCAGCTGGCCTATTA	ACTGCTTCAACTGTGCATGA
DAO	GTTCCTAATCTCGGGCTACAAC	AACCAGCCATAGCCGTAATC
DDO	CTGACGTGGTTCTGGGATTT	GGCATTCACATTTCAGGGTTG
DECR2	ATACGGTGATTGCCAGTAGG	TCGGACGTCCATAGAGAGAG
DNAJC10	CTTCAGTGGTCTCCCTTACAC	GATGACACCACGGAGAATAGAA
DNM1L	GCAAAGGATCATTCAGCACT	CAGGCAACCTTTTACGAAGA
ECH1	AAGAGGAATGCCATGAACAA	AACATTTTTCCTGCACCAGA
EHHADH	TCTAGAAAGGGGCAAGGTCT	TTCACAGAGCACATCAGGAA
EPHX2	CATCTGCTCCTCCCGAAATAG	CTTGAGAGAGGCCCAGTTTATC
FAR1	GAGCTACCGGTTTTCTAGGG	GTGGTGTCTGTCCAGCTTTC
FAR2	AGCAACAGCTTCACATCACA	TTAAAAGCCGATTCATGAGC
FIS1	GACAAGGCCATGAAGAAAGA	GGATTTGGACTTGGACACAG
GNPAT	TTTTTGTGCGCCCATCCTTAG	AAAACATCACGTAGGAAGCGAAAG
GSTK1	CCTGTGCCGGTATCAGAATATC	GAGGCTTGTTTCCACTGTCT
HACL1	ATGCTGGTACTTTCGGAACA	GAAAACCCAAATGCACTGTC
HAO1	AGGCAGAGAAGATGGGCTACAA	TTTTCATCCTGAGTTGTGGCGG
HAO2	GGAGATCAGTGCCCCTATTT	GCAAATGTGCTGGTGATGTA
HMGCL	AAGGGCATTCAGAAGTTTCC	GGCAGCTCCAAAGATGACTA
HSD17B4	GGCTTTGTCACGAGAGTTGT	CAGGAGTCATTGGGTGATTC
IDE	CAAGTCAGCTGGTTCGGTATAG	GCTTTGCATGTCTGTTTGGTAG
IDH1	CTAAGGGTTGGCCTTTGTATCT	GGGACTTGTACTGCTTGTCATA

IDI1	CACCGAAAATAAGCTTCTGC	GCTGGATTGCTTAATGGATG
IDI2	AGTCACGTTTCCTGGGTATTT	CACTCCGATGGCATCCTTT
MLYCD	ACATCCAGGCAATCGTGAAG	CTGGGTCAAGCTGATGGAATAA
Mosc2	ACCTGGGATGAACTCCTAATTG	AGTGGCTGTTTCCTGTCTATG
MPV17	GCCAAACTACAGCGGGATTA	GGACAGGTAGGAGTTCCAGATA
MVK	AGAGCAAGTGGAGAAGCTAAAG	CGGCAGATGGACAGGTATAAG
NOS2	GTCAGAGTCACCATCCTCTTTG	GCAGCTCAGCCTGTACTTATC
NUDT12	AGGAGGAAGATGGATTGGTTGCCT	CAGAAGGGCTGGCATAGGAGGA
NUDT7	CTAAGGCCCGCTTAAGAAAGTA	CGGACGGTGAACAACAAATG
PAOX	GGGAGTACCTCAAGAAGGAGAT	CCAGGTTGAAGAAGGAGTTCAG
PECI	GACAGGGCAACATTTCATACAC	CTTGGCTGGGCTCATTATCT
PECR	CTTGGTGAACAATGGAGGAG	GGAGCTGTAAACTGCTTTGC
PEX1	TTCCTTAGGCGTATCCTCCTT	ATTCCTAAGTCCTGCAACAAGAG
PEX10	CTCTCAGATGTGGCCTACTTT	CTGGATGATGCTGACGTACT
PEX11A	GCTGCTCACCACTACTACTATTC	CCTGGGATGCTGATTTCTCTT
PEX11B	TGCTAGACGTGGTCAGAAATG	CCAGGGATAGATTAGGGTGAGA
PEX11G	GTTGGTGGAGTTCTGGTTGA	GGTCATCAAAGAGTCGCAAGA
PEX12	CTTGCAGTTCCTTGACTGGT	TTGGGTAAGAGGGGAGAATC
PEX13	TGGTCCTTACCTCATTTGGA	ACGGCAGCAAAATCATATTC
PEX14	CCCTCACCTCATATCTCAGC	GGGGAGCAGGTATTTCTTGT
PEX16	GAGCCTCCTGAGTGACAGAA	GCGGTCATAGAAAGGAGAGC
PEX19	GGAGAGTGGGCAGTGATATG	CATGCTGGAGTTCTGAAGGT
PEX26	TGGATGTACTTCAGGCCATT	ACAGGAACTTGTGGGAGACA
PEX3	GAAATCTCGTTGAGCAGCAT	CCTGCACTGCTAATGGAGTT
PEX5	TACAGCAGCAGGGTACATCA	CCCAGAAATCGACATCAGAC
PEX5L	GTTCCACCTGAGTGGAGAATTTA	GAGGCGGTTCCATAGTGAATAG
PEX6	AAACTGCAGGCCATCTTCTCCCGG	AGCCATCACACGGGCATCCTCA
PEX7	CATGTCCTCATCACCTGTAGTG	CCTCCTGAGCGTGTTCTTTAT
PHYH	GCTTTCGGAATGAGTTTGAA	TGGACCTTCGTGATCATCTT
PIPOX	GAGAGAAGGTGGTGGAGATAAAC	GCTGTGATGACCAAGCTCTTA
PMVK	TCAGCGGCAAGAGGAAATC	CCTGAGCATACTGTTCCTTGAG
PRDX1	CGCACCATTGCTCAGGATTA	CCAACAGGGAGGTCATTTACAG
PRDX5	GGATGTTCCAAGACACACCT	CCAGTCACAAAGGCATCATT
PXMP2	TTTGTCAGCACTTGGGAACT	CCTGTGAAGAAGAACCCGTA
PXMP3	GAATGCGAAGAGTGCAAACAG	CTGAGTAAACTGGGACCAAACT
PXMP4	TGCCCTGCAGTCCTACATAC	TGTTGATCTGGCTGTTGATG
RHOC	TGTCATCCTCATGTGCTTCTC	CTTGCCTCAGGTCCTTCTTATT
RN18S	CTGAGAAACGGCTACCACATC	GCCTCGAAAGAGTCCTGTATTG
SCP2	AGGGAAGCCTTGGAATAAAA	TCCAGCATACCCAAACATCT
SLC22A5	CTCTCAGGGACGATTTGAAGAG	CTCACTCGGGTCAAAGATAGTG
SLC25A17	CTGCTGCTCCAATTTTGTCT	CCTGCAACAAACCCAACTAC
SLC27A2	CTCTTGCCTTGCGGACTAAA	CCGAAGCAGTTCACCGATATAC
SOD1	GTGCAGGGCATCATCAATTTC	GGCCTTCAGTCAGTCCTTTAAT
SOD2	GGAGATGTTACAGCCCAGATAG	CGTTAGGGCTGAGGTTTGT
TRIM37	GGGACCAGCATTGGTACATTA	CTGAGTTCGAGACAGCTCAATAG
XDH	AGCCTCTCGCCATCTTTATTC	TCTTTCAGCCTCAGCAACTC

Supplementary Table 2. qRT-PCR primer used in this study.

Supplementary Table 2

Genes for expression analysis	Forward primer sequence	Reverse primer sequence
hADAMTS4	CGCTTTGCTTCACTGAGTAGAT	CTGTTAGCAGGTAGCGCTTTAG
hADAMTS5	GCACTGGCTACTATGTGGTATT	AGCCAGTTCTCACACACTTC
hCOL2A1	TGGACGCCATGAAGGTTTTCT	TGGGAGCCAGATTGTCATCTC
hMMP13	TTGCAGAGCGCTACCTGAGATCAT	TTTGCCAGTCACCTCTAAGCCGAA
hPGAM1	CATCAGTAAGGATCGCAGGTATG	CTTCATTCCAGAAGGGCAGAG
hRN18S	CTGAGAAACGGCTACCACATC	GCCTCGAAAGAGTCCTGTATTG
mADAMTS4	GAGCTGTGCTATTGTGGAAGA	CCCTGCCCATTCAAGTTAGT
mADAMTS5	CTCGATCCCTAGCTGTCTTTG	CAGGAGTGGCTTTAGAGTGTAG
mIL-1β	CCACCTCAATGGACAGAATATCA	CCCAAGGCCACAGGTATTT
mIL-6	CCAGAGTCCTTCAGAGAGATACA	CCTTCTGTGACTCCAGCTTATC
mMMP13	CCCTGATGTTTCCCATCTATACC	TTCATCGCCTGGACCATAAAG
mMMP9	ATTACAGGGCCCCTTCCTTA	CCACATTTGACGTCCAGAGA
mPGAM1	CCCTTCTACAGCAACATCAG	CTGGCAATAGTGTCCTTCAG
mTNF-α	TTGTCTACTCCCAGGTTCTCT	GAGGTTGACTTTCTCCTGGTATG
mRN18S	CCAGTAAGTGCGGGTCATAAG	GGCCTCACTAAACCATCCAA
mCOL2A1	CTGGTTTGGAGAGACCATGAA	GAGGAAAGTCATCTGGACGTTAG
ChIP mPGAM1-1	TTACAAGTGAGGCTGAGACG	CCCTGCGCAATTCCAATATAC
ChIP mPGAM1-2	CTGAAGAACTGGTCAGAGGATAG	GCGCACGTAAGCAAGGA
ChIP mACTB	CGTATTAGGTCCATCTTGAGAGTAC	TATTGCCATTGAGGCGTGATCGTAGC
